# The Oligomerization Domains of the APC Protein Mediate Liquid-Liquid Phase Separation That Is Phosphorylation Controlled

**DOI:** 10.3390/ijms24076478

**Published:** 2023-03-30

**Authors:** Shachar G. Bressler, Amit Mitrany, Alon Wenger, Inke Näthke, Assaf Friedler

**Affiliations:** 1 The Institute of Chemistry, The Hebrew University of Jerusalem, Edmond J. Safra Campus, Givat Ram, Jerusalem 91904, Israel; 2 Division of Molecular Cell and Developmental Biology, University of Dundee, Dundee DD1 5AA, Scotland, UK

**Keywords:** intrinsically disordered proteins, adenomatous polyposis coli, liquid-liquid phase separation, post translational modifications, phosphorylation

## Abstract

One of the most important properties of intrinsically disordered proteins is their ability to undergo liquid-liquid phase separation and form droplets. The Adenomatous Polyposis Coli (APC) protein is an IDP that plays a key role in Wnt signaling and mutations in *Apc* initiate cancer. APC forms droplets via its 20R domains and self-association domain (ASAD) and in the context of Axin. However, the mechanism involved is unknown. Here, we used peptides to study the molecular mechanism and regulation of APC droplet formation. We found that a peptide derived from the ASAD of APC-formed droplets. Peptide array screening showed that the ASAD bound other APC peptides corresponding to the 20R3 and 20R5 domains. We discovered that the 20R3/5 peptides also formed droplets by themselves and mapped specific residues within 20R3/5 that are necessary for droplet formation. When incubated together, the ASAD and 20R3/5 did not form droplets. Thus, the interaction of the ASAD with 20R3 and 20R5 may regulate the droplet formation as a means of regulating different cellular functions. Phosphorylation of 20R3 or 20R5 at specific residues prevented droplet formation of 20R3/5. Our results reveal that phosphorylation and the ability to undergo liquid-liquid phase separation, which are both important properties of intrinsically disordered proteins, are related to each other in APC. Phosphorylation inhibited the liquid-liquid phase separation of APC, acting as an ‘on-off’ switch for droplet formation. Phosphorylation may thus be a common mechanism regulating LLPS in intrinsically disordered proteins.

## 1. Introduction

Intrinsically disordered proteins (IDPs) and intrinsically disordered regions in proteins (IDRs) constitute up to 70% of the proteome [1]. IDPs take advantage of their flexibility to form multiple protein–protein interactions (PPI) that can change readily and are easily regulated. IDPs serve as scaffold proteins and assemblers, and are abundant in many cellular pathways, such as signal transduction, where multiple PPI are required. IDRs are known as preferred sites for protein phosphorylation [2,3]. Many IDPs also tend to undergo a liquid-liquid phase separation (LLPS), a phenomenon by which proteins assume droplet-like formations [4,5,6,7]. Unlike oil droplets or aggregates, droplets of biomolecules contain large amounts of water, conserving the biological activity of macromolecules that reside within them and allowing diffusion into their cores. For example, droplets of RNA increase the cleavage and ligation of RNA by the Hammerhead ribozyme. In addition, droplets increase the activity of enzymes such as Hexokinase and pyruvate kinase [8,9,10]. In cells, LLPS of biomolecules such as IDPs and RNA produces membrane-less organelles, that are used, for example, as stress granules, P-bodies and Lewy bodies [11,12,13]. Post transcriptional modifications (PTMs) of proteins, particularly phosphorylation, can affect the LLPS of IDPs. For example, Tau and FUS proteins form droplets upon phosphorylation [14,15].

**Figure 1:**
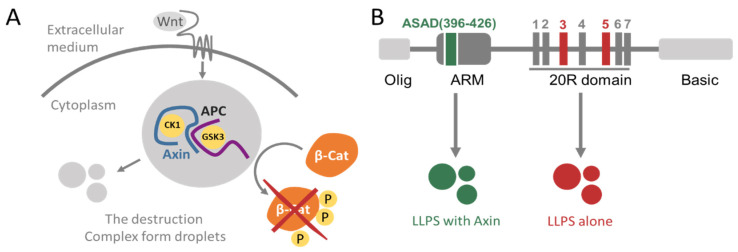
Schemes of Wnt signaling and the domain structure of APC (**A**) a scheme of Wnt signaling. When active, the destruction complex, composed of the scaffold proteins APC and Axin along with kinases CK1 and GSK3-β, phosphorylates, and thus targets, β-Catenin for degradation by the proteasome. Components of the destruction complex, particularly Axin, have been described as forming droplets. (**B**) Domain structure of APC. From left to right: the oligomerization domain of APC, the ASAD at the end of the ARM repeats (green), the 20R domain that contains seven repeats, 20R1 to 20R7, with 20R3 and 20R5 labeled (red), and the basic domain of APC. The ASAD undergoes LLPS with Axin, while the 20R domains form droplets by themselves [28].

The tumor suppressor Adenomatous polyposis coli protein (APC) is a 2843-residue (in humans) IDP (numbering of amino acid residues will refer to human APC throughout the manuscript, unless noted otherwise). Mutations that cause truncation of APC are responsible for 80% of sporadic colon cancers [16,17,18,19]. APC is mostly disordered, and its structured domains reside in the N-terminal third, most notably the Armadillo repeat (ARM) domain between residues 340–731. This domain is composed of at least six (depending on specific APC proteins) repeating units each comprising three α-helices [20]. APC oligomerization can occur both by self-interactions and by associating with binding partners. It forms a dimer via N-terminal self-interactions [21]. Moreover, the domain, including residues 782–1018, binds to itself and to the extreme C-terminal domain, regulating the dynamics of APC clusters at microtubule ends [22]. APC is a multi-functional protein with a key role in the Wnt signaling pathway. It also directly and indirectly binds to and regulates cytoskeletal proteins, contributing to other cellular processes such as cell migration, cell death and mitosis [23,24,25]. When Wnt signaling is inactive (i.e., in the absence of a Wnt ligand), the so-called β-catenin ‘destruction complex’ is active. This complex contains, among other proteins, APC, Axin, and the kinases CK1 and GSK3-β. APC and Axin are the scaffold proteins that assemble the destruction complex. They bind CK1 and GSK3-β, facilitating the phosphorylation of β-catenin, targeting it for proteasomal degradation [26,27] (Figure 1A). In the presence of Wnt ligand, the destruction complex is inactive, allowing β-catenin to increase and act as a transcriptional regulator driving expression of Wnt target genes to, for instance, stimulate proliferation.

APC contains several unstructured domains that bind different proteins (Figure 1B). For example, APC can oligomerize via its self-association domain (ASAD) at residues 396–426, located near the beginning of the ARM domains and via its oligomerization domain (residues 1–54, named Olig) that is responsible for its dimerization [28]. The C-terminus of APC at residues ~2200–2400, termed the basic domain, binds microtubules (MT) and this is important for cell migration and division [29]. APC undergoes post-translational modifications (PTMs), in particular phosphorylation, by the kinases GSK3-β and CK1, mostly in the serine-rich domain of APC (residues ~1260–2000). The serine-rich domain contains seven conserved repeats, known as the 20R1-7. The CK1 and GSK3-β kinases phosphorylate the 20R domains at different residues, resulting in different phosphorylation patterns that lead to different biological outcomes. CK1 can phosphorylate the 20R domains at several serine residues. It phosphorylates 20R3 at serines 1501, 1504, 1507, and 1510. Phosphorylation of serine 1505 by CK1 is a prerequisite for phosphorylation of serine 1501 by GSK3-β. Furthermore, CK1 phosphorylates 20R5 at serine 1861 and 1863. Phosphorylation of serine 1861 by CK1 is a prerequisite for phosphorylation at serine 1857 by GSK3-β [30,31]. Phosphorylation of APC is an important regulatory mechanism of its cellular functions. When phosphorylated by CK1 and GSK3-β, the 20R3 repeat binds β-catenin significantly tighter than the non-phosphorylated 20R3, leading to β-catenin degradation [30,32,33]. Non-phosphorylated APC, on the other hand, binds better to MTs, enabling its stabilizing effect which is important for migration and division [34,35,36,37,38].

The ability of APC to undergo LLPS involves at least two domains, ASAD and the 20R domain, and APC forms droplets together with Axin [28]. APC2 is one of the two APC proteins expressed in *drosophila*. It is significantly shorter than human APC with only 1067 amino acids. In APC2, the N-terminal coils preceding ASAD in human APC are lacking so that ASAD is much closer to the N-terminus of the protein and it contains a slightly different sequence. For APC2, ASAD is an essential domain that affects the size of droplets [28]. Co-expression of Axin with APC2 lacking ASAD produced smaller droplets than those formed when wild-type A PC2 was expressed in cultured human cells [39]. This was observed only when APC2 was co-expressed with Axin, but not for APC2 alone. Moreover, when 20R domains of human APC undergo LLPS the probability of droplet formation increases dramatically with their number [40]. However, the mechanism of droplet formation by APC, the precise residues involved, and how this process is controlled, are unknown.

Here we used peptides derived from different domains of the human APC protein to study the molecular mechanism for its LLPS. We identified short peptides representing key APC domains that undergo LLPS and used them to reveal the molecular mechanism responsible for the formation of droplets and its regulation. The ASAD adopted a helical secondary structure and formed droplets via hydrophobic interactions. Using peptide array screening we found that the ASAD binds the 20R3 and 20R5 domains of APC. Peptides derived from these two 20Rs formed droplets on their own and their droplet formation was inhibited by phosphorylation at sites known to be phosphorylated by the kinases-regulating APC activity. Phosphorylating the 20Rs at the GSK3-β kinase target residues, p20R3 (pS1501, 1505) and p20R5 (pS1857, 1861), prevented droplet formation. Similarly, when 20R3 was phosphorylated in the phosphorylation pattern produced by the kinase CK1, p20R3 (p1504, 1507), it also did not form droplets. On the other hand, phosphorylation of 20R5 with the corresponding pattern, p20R5 (pS1863) did not inhibit its ability to form droplets. We found that the sub-domain S_1_-L_2_-S_3_-S_4_-L_5_ of the 20Rs is essential both for binding ASAD and for regulating droplet formation. Alanine scanning of the sub-domain S_1_-L_2_-S_3_-S_4_-L_5_ revealed that the third serine (Ser3) was essential for regulating droplet formation. This repeat is found in all seven 20R regions. We conclude that this repeat is a ‘sticky motif’ that allows the 20R domain to undergo LLPS.

Our results reveal that 20R3 and 20R5 peptides, both intrinsically disordered, have a significant role in the oligomerization of APC. They undergo LLPS and bind the ASAD via the SLSXL repeat, thus participating in various oligomeric forms of APC. Phosphorylation of the 20Rs regulates their LLPS and it is known that phosphorylation of SLSXL promotes the binding of APC and E-cadherin to β-catenin [32,41]. We conclude that phosphorylation of 20R3 and 20R5 is a “on/off” switch for LLPS that can also regulate the biological function of APC via the SLSXL domain of the 20Rs.

## 2. Results

### 2.1. The ASAD Binds Itself and Forms Droplets via Hydrophobic Interactions

**Figure 2:**
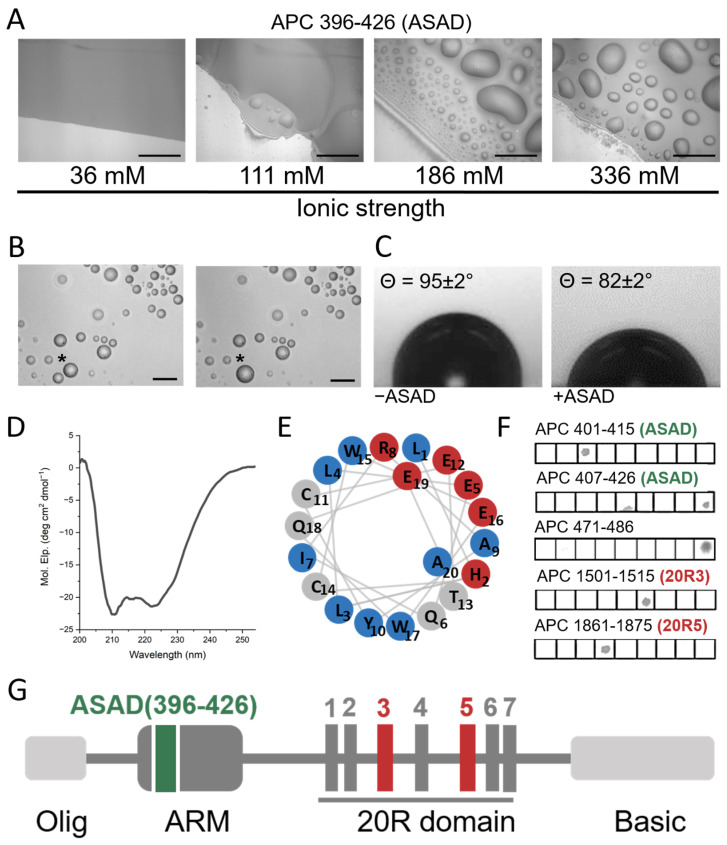
The ASAD binds itself and forms droplets via hydrophobic interactions. (**A**) The ASAD forms droplets in an ionic-strength-dependent manner. All images were taken after 5 min of incubation and show fusion of the droplets (Figure 2B). Scale bar = 20 µM. (**B**) A fusion event between two droplets. Fusion of droplets was monitored for the p20R5CK1 peptide undergoing LLPS. Shown are droplets of p20R5CK1 before (left) and 6 s after (right) they fused to form a larger droplet. The droplets before and after fusion are labeled by (*). Scale bar = 20 µm. (**C**) contact angle experiments of the ASAD on a glass slip covered by parafilm show that addition of the ASAD creates a more hydrophobic environment in the solution. (**D**) CD spectra show that the ASAD is α-helical. (**E**) A wheel projection of the ASAD shows that it forms a charged patch on one (red circles) and hydrophobic patch (blue circles) on the other side of the α-helix. (**F**) Peptide array screening of the ASAD binding to APC peptides shows that the ASAD binds itself, to helix 2 in the ARM domain it is part of and to two 20Rs domains (20R3 and 20R5). The spot on the 5th square on APC407-426 (ASAD) line is an artifact, caused by bleeding from the signal in the square below (which is not shown). (**G**) Domain structure of the APC protein. With the position of the ASAD and 20R3/5 indicated in green and red respectively.

To determine the role of the ASAD in the LLPS of APC, we examined whether the ASAD could form droplets by itself. A peptide corresponding to the ASAD (APC 396–426) formed droplets as observed by differential interference contrast (DIC) microscopy, while fusion events of the droplets were monitored to confirm LLPS (Figure 2B). Increasing ionic strength enhanced the droplet formation of the ASAD. At an ionic strength of 336 mM, the ASAD rapidly formed a significant number of droplets, while at an ionic strength of 36 mM, it did not (Figure 2A). This suggested that ASAD droplet formation occurs via hydrophobic interactions. Upon adding the ASAD to buffer solutions on a hydrophobic surface, the contact angle was 82 ± 2° compared to 95 ± 2° for buffer alone, indicating that the addition of the ASAD enhanced the interactions of the solution with the hydrophobic surface, showing that the solution was more hydrophobic [42] (Figure 2C).

Circular dichroism (CD) studies showed that the ASAD forms an α-helical secondary structure with typical minima at 210 nm and 225 nm (Figure 2D). A helical wheel projection of the crystal structure of the 19 residues peptide derived from the ASAD (PDB 5IZA) [20] showed an amphipathic helix, with the charged residues His2, Glu5, Arg8, Glu12, Glu16, and Glu19 located on one side, creating a charged patch, while the hydrophobic residues Leu3, Ile7, Tyr10, and Trp17 form a hydrophobic patch (Figure 2E). These findings indicate that the secondary structure of the ASAD plays a part in its droplet formation, and the ASAD may form droplets in solution to decrease the exposure of the hydrophobic residues to the polar solvent.

**Figure 3:**
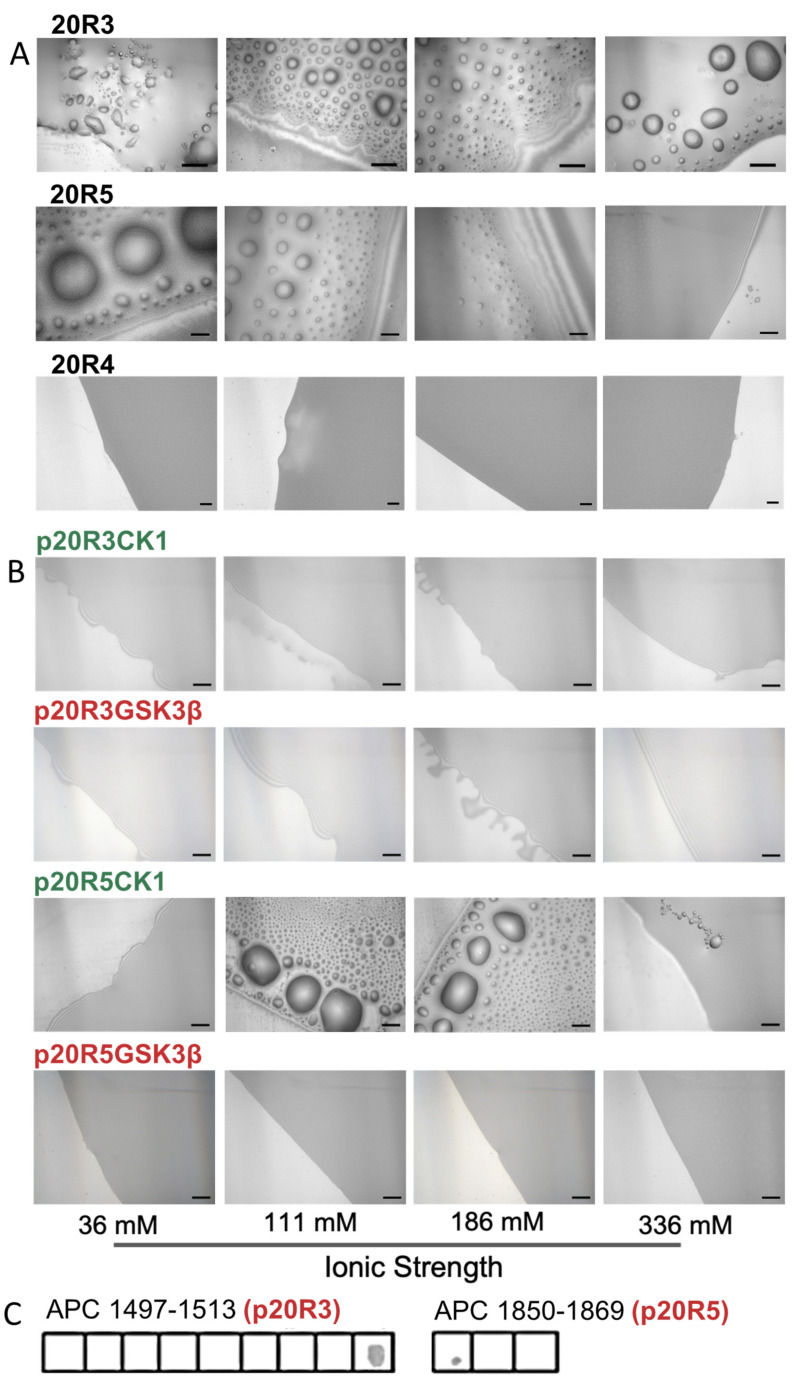
Phosphorylation prevents droplets formation by 20R3 and 20R5. (**A**) DIC microscopy imaging shows droplets formed by 20R3, 20R5 but not 20R4 at different ionic strength conditions. (**B**) Effect of phosphorylation on droplet formation. Peptides with the phosphorylation patterns of the kinases GSK3-β, p20R3GSK3-β, and p20R5GSK3-β (red) did not form droplets, while p20R5CK1 with a phosphorylation pattern of the kinase CK1 (green) did. 20R4 did not form droplets at any ionic strength. Scale bar = 20 μm. (**C**) Effect of phosphorylation on ASAD binding to 20Rs. Peptide array screening showed binding of the ASAD to phosphorylated 20R domains when phosphorylated in the GSK3-β. pattern.

Our next aim was to determine whether additional domains in APC are involved in its droplet formation by first investigating whether other domains in APC bind to the ASAD. We used peptide array screening to identify domains in APC that can bind the ASAD_396–426_ (Figure 2F). The ASAD peptide was incubated with an array of 15 residue-long peptides, with a five-residue overlap, which covered the full-length sequence of the human APC protein. We found that the ASAD bound peptides representing three regions of APC: the ASAD itself (i), and two 20R domains (ii) and (iii). The ASAD bound the α-helical peptides APC_407–426_ and APC_401–415_ (i), located at the N-terminus of the solvent-exposed Armadillo-like (ARM) repeat (Figure 2A) and part of the ASAD itself, confirming previous observation [22]. The ASAD did not bind APC_391–405_ and APC_411–425_ (Table S1), which are adjacent to APC_401–415_ and share residues 401–405 and 411–415. This suggests that the most important residues mediating ASAD binding are ^406^VLHLL^410^. This sequence contains mostly hydrophobic residues, consistent with the idea that the ASAD binds itself via its hydrophobic patch; APC_1501–1515_ (ii) is part of the 20R3 domain. The ASAD bound a peptide derived from 20R3, APC_1501–1515_, but did not bind the adjacent peptides on the peptide array, APC_1491–1505_ and APC_1511–1525_. This result suggests that the domain of 20R3 that binds the ASAD is ^1506^SLSAL^1510^ (Table S1). The ASAD bound APC_1861–1875_ (iii), which is derived from the 20R5 domain. The ASAD did not bind the adjacent peptides APC_1851–1865_ and APC_1871–1885_ suggesting that the ASAD binds the 20R5 domain via ^1866^SLSSL^1870^ (Figure 2G and Figure 3A). In summary, the ASAD bound ^1506^SLSAL^1510^ and ^1866^SLSSL^1870^. Both share an almost identical sequence. This sequence pattern, S-L-S-X-L, with X representing serine, alanine or aspartic acid, is found in all seven 20R repeats in APC.

### 2.2. 20R3 and 20R5 form Droplets in an Ionic-Strength-Dependent Manner

Since 20R3 and 20R5 bind the ASAD, we tested whether the 20R peptides also contribute to the LLPS of APC, either by forming droplets by themselves and/or together with the ASAD. Both 20R3 and 20R5 formed droplets on their own (Figure 3B). 20R5 formed droplets at an ionic strength range of 36–186 mM, while 20R3 formed droplets at ionic strengths of 36–336 mM. Increasing the ionic strength led to the formation of fewer droplets, with larger diameters. A peptide corresponding to the 20R4 domain, which did not bind the ASAD in the peptide array, also did not form droplets at any ionic strength.

### 2.3. LLPS of the 20R Peptides Is Regulated by Phosphorylation and Is Kinase Dependent

Next, we examined whether phosphorylation, a known PTM of APC in cells and an important regulation mechanism for LLPS, regulates droplet formation of the 20Rs. We examined if and how specific phosphorylation patterns corresponding to those of APC-phosphorylating kinases affected the droplet formation of the 20R peptides. We synthesized the 20R3 and 20R5 peptides with phosphorylated serine at sites known to be phosphorylated by these kinases in cells (Figure 3A). The peptides with the phosphorylation patterns for GSK3-β were p20R3GSK3-β and p20R5GSK3-β and the peptides with the phosphorylation patterns of CK1 were p20R3CK1 and p20R5CK1 [20] (Table 1), and their droplet formation was tested at several ionic strengths (Figure 3C). Neither p20R3GSK3-β nor p20R5GSK3-β formed droplets at any ionic strength. p20R5CK1 formed droplets at an ionic strength close to physiological values, suggesting that 20R5 phosphorylated by CK1 can form droplets in cells. However, at a lower ionic (36 mM) and higher ionic strength (336 mM), it did not form droplets. The p20R3CK1 peptide did not form droplets at any ionic strength.

### 2.4. The ASAD and 20R Peptides Do Not form Droplets When Co-Incubated

**Figure 4:**
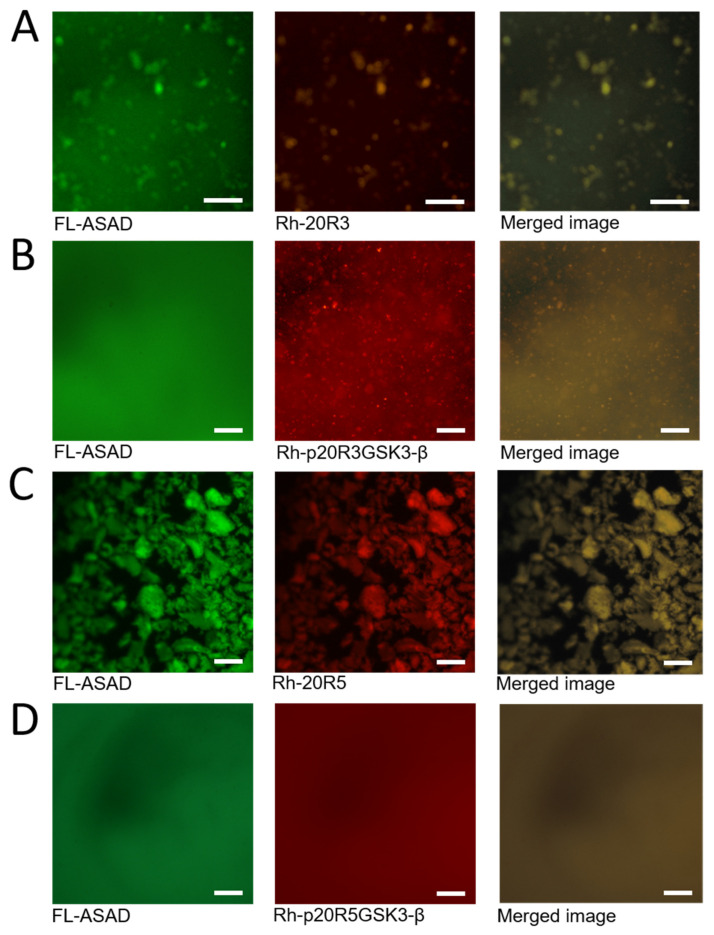
The effect of phosphorylation on droplet formation by APC-derived peptides. (**A**) The ASAD labeled with fluorescein (green) and 20R3 labeled with rhodamine b (red) are located at the same clusters (yellow) (**B**) Phosphorylation of 20R3 prevented co-aggregation with the ASAD. (**C**) ASAD labeled with fluorescein (green) and 20R5 labeled with rhodamine b (red) located at the same clusters (yellow) (**D**) Phosphorylation of 20R5 prevented co-aggregating with the ASAD. All scale bars = 20 μm.

Using peptide arrays we discovered that the ASAD bound peptides derived from 20R3 and 20R5 domains even when they were phosphorylated in the GSK3-β phosphorylation pattern (Figure 4A). To understand the relationship between the interaction of the ASAD and the 20Rs and their ability to form droplets, we incubated the fluorescein-labeled ASAD peptide (FL-ASAD) with rhodamine B-labeled 20R peptides (Rh-20R3 and Rh-20R5). We detected aggregates of FL-ASAD in the presence of both Rh-20R3 and Rh-20R5 (Figure 4B,D). Unlike liquid droplets, these aggregates were not round and did not show the Brownian motion characteristic of LLPS droplets. In addition, we did not detect their fusion with each other. These observations support the conclusion that the ASAD and the 20Rs do not form droplets that result from LLPS when bound to each other. Merged images showed that the ASAD and the 20Rs co-localized in the same aggregates (Figure 4A,C, respectively). Together with results from the peptide array, this indicates that the ASAD and the 20Rs bind each other and co-aggregate. Next, we measured how the phosphorylation of the 20Rs affected their behavior when incubated with the ASAD. The ASAD was incubated with p20R3GSK3-β and p20R5GSK3-β. The phosphorylation of both 20Rs prevented co-aggregation with the ASAD, although small structures were still visible for p20R3GSK3-β (Figure 3B,D).

### 2.5. The SLSXL Repeat within the 20R Peptides Binds the ASAD and Is Responsible for Droplet Formation of 20R3 and 20R5

**Figure 5:**
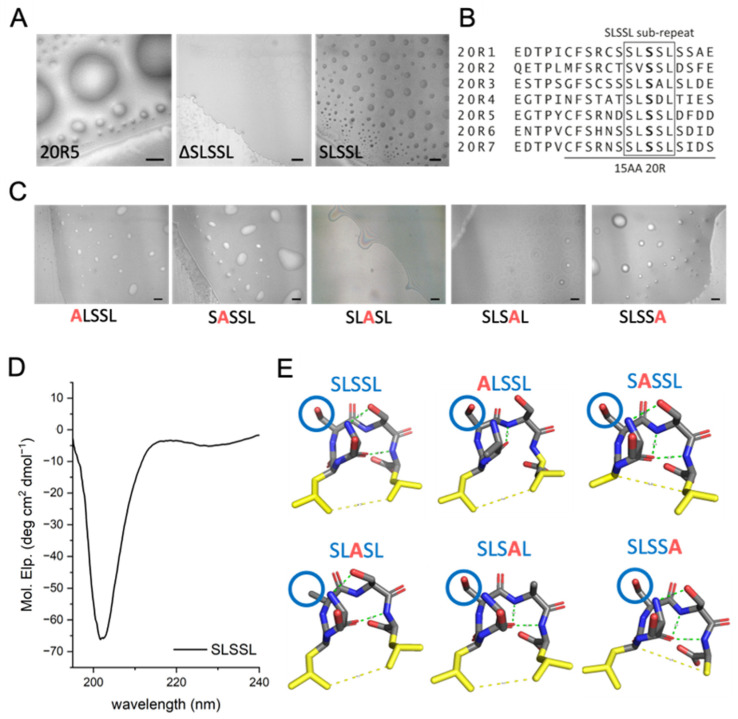
The SLSSL sequence, and particularly the 3rd serine in the sequence, are essential for LLPS of the 20R peptides. (**A**) Imaging of 20R5, SLSSL, and ΔSLSSL using DIC microscopy reveals an important role for SLSSL in droplet formation of 20R5. Scale bar = 20 μm. (**B**) Sequence alignment of all the 20R peptides show that the SLSSL, especially the 1st and the 3rd serines, are present in all 20R repeats. (**C**) Alanine scan of SLSSL at an ionic strength of 36 mM showed that ALASL, and SLSAL did not form droplets, while ALSSL, SASSL, and SLSSA formed droplets. Scale bars = 20 µm. (**D**) CD spectrum of SLSSL sub-repeat shows that it has a minimum at 203 nm, suggesting it forms a β-turn secondary structure. (**E**) Predictions from PEP-FOLD3 [43] show that all of the SLSSL peptides produced by alanine scanning are predicted to have a β-turn secondary structure. The 3rd serine is labeled with a blue circle.

We found that the SLSXL sequence in 20R3 and 20R5 is essential for binding the ASAD. Therefore, we tested whether it is also involved in droplet formation. We selected 20R5 as a model for LLPS experiments since it contains the SLSSL repeat, which is the most common variation of the SLSXL repeat within all seven 20R domains (Figure 5B). We synthesized 20R5 without the SLSSL repeat (ΔSLSSL) and found that it did not form droplets (Figure 5A). This indicates that the SLSSL sequence is essential for droplet formation by 20R5. We then synthesized a peptide corresponding to the SLSSL repeat and tested its ability to undergo LLPS. The SLSSL peptide on its own formed droplets at an ionic strength of 186 mM (Figure 5A).

To identify the precise residues in SLSSL that mediate droplet formation, we performed alanine scanning of SLSSL. The peptides ALSSL, SASSL, and SLSSA all underwent LLPS at an ionic strength of 36 mM, while SLSAL formed only few droplets. Moreover, SLASL did not form droplets at all (Figure 5C). These results suggest that the 3rd serine has a crucial role in mediating the SLSXL droplet formation. CD spectroscopy of a peptide corresponding to the SLSSL sub-repeat has a minimum at 203 nm, indicating it forms a β-turn (Figure 5D). This correlates with predictions from PEP-FOLD3 [43], suggesting that the SLSSL repeat forms a hairpin (Figure 5E).

## 3. Discussion

LLPS of many proteins, particularly IDPs, provides versatile regulation of their interactions and functions. The tumor suppressor protein APC is an example of an IDP with multiple cellular functions that have to be coordinated for normal tissue homeostasis. APC plays a key role in Wnt signaling and it has been proposed that this is regulated by LLPS [28]. Similarly, clustering of APC at the end of microtubules and microtubule bundles is important for its contribution to cytoskeletal regulation [37,38,44]. However, the mechanisms and regulation underpinning the formation and functions of these structures and the relationship between them are not understood. Here we used a peptide approach to gain insight into the specific regions of APC that mediate its LLPS and to understand the regulation of this process by phosphorylation. Working at the peptide level also allowed us to compare the effect of phosphorylation in specific and different phosphorylation patterns. Indeed, we identified different domains and specific residues within them that can contribute to the process of droplet formation of APC. This provides a novel mechanistic understanding of how APC can adopt a distinct biophysical state that is involved in its function. We found that self-association of at least three distinct domains contribute to LLPS: the ASAD, 203R, and 20R5. In addition, the ASAD can bind to 20R3 and 20R5. However, this interaction leads to the loss of their ability to undergo LLPS. Instead, in combination, ASAD and either 20R3 or 20R5 form aggregates with different appearance and dynamics rather than droplets. We propose that the interactions and their regulation by phosphorylation that we discovered and have described here are core mechanisms for regulating the recruitment of APC into a pool that is primed to regulate Wnt signaling. The fact that cytoskeletal regulation in APC and its role in Wnt signaling appear to be mutually exclusive suggests that APC in LLPS is unavailable to interact with cytoskeletal proteins [45,46].

### 3.1. A peptide Approach Reveals New Oligomerization Domains in APC

Previously, LLPS was reported as a unique property of disordered proteins and 50- to 100-residues-long oligopeptides [47,48]. It is difficult to follow LLPS at the protein level since it is usually difficult to obtain large proteins for in vitro studies. Large proteins tend to undergo non-specific aggregation and they are prone to proteolysis. Furthermore, examining the role of specific PTMs is challenging, since kinases tend to phosphorylate non-specific sites in vitro, and substitution of phosphate groups with glutamic or aspartic acids mimics neither the charge nor the size of phosphate group accurately [49]. Thus, working with whole proteins is not always possible and often does not allow pinpointing of the exact residues that are the key for oligomerization and LLPS [48,50]. Here, using APC, we demonstrate that working with peptides can overcome these limitations and allows us to identify the precise residues responsible for oligomerization and LLPS of a large, multifunctional protein that is a major tumor suppressor. This approach revealed the specific domains of the ASAD required for LLPS, and the regulatory mechanisms for the formation of different assembly states with different biochemical properties and biological functions.

**Figure 6:**
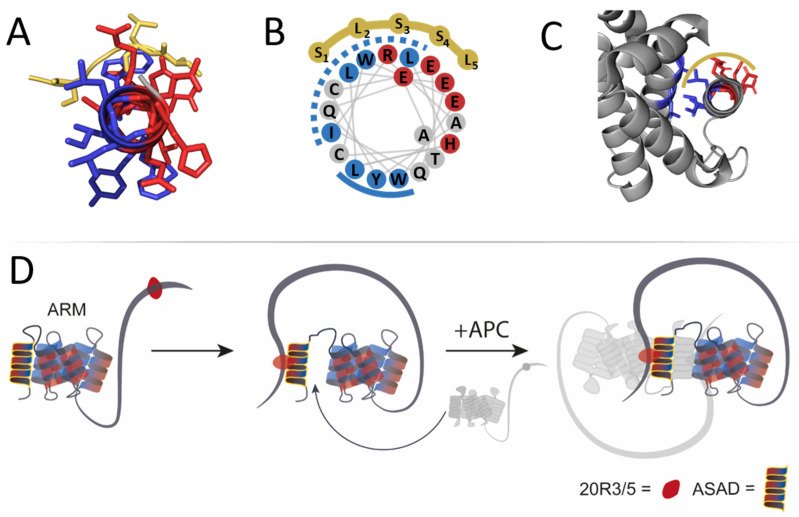
A scheme of the proposed self-association process of APC mediated by residues ~350–2000 spanning the ARM and 20R domains. (**A**) A prediction of the ASAD (red and blue helix) binds the SLSSL repeat (dark yellow) via both hydrophobic and charged patches. (**B**) A wheel projection of the ASAD, showing its two hydrophobic patches, 1 and 2 (dashed and solid blue lines, respectively). SLSSL repeat binds the ASAD via its first hydrophobic patch and the charged patch, possibly promoting separation of the ASAD from the ARM domain. (**C**) Crystal structure [20] of the ARM repeat (gray) shows that the ASAD (red and blue helix) binds the following helical repeat by its second hydrophobic patch, which does not bind SLSSL. (**D**) 20R3/5 (red circles represent either 20R3, 20R5, or both) binds the ASAD (yellow). Upon binding SLSSL, the first hydrophobic patch (dashed line, Figure 6B) dissociates from the ARM repeat. The disassociation of the second hydrophobic patch of the ASAD (solid line, Figure 6B) is required for APC oligomerization by binding the ASAD from another APC molecule (light gray).

We found that in addition to oligomerization via previously described sites [21,22,28,51], APC can also undergo oligomerization via its ASAD and 20R domains, which could regulate its role as a scaffold protein in the destruction complex. Our results regarding the binding sites of the ASAD for other domains of APC are consistent with the crystal structure [20] data and alphaFold predictions of the APC ARM domain [52]. The ASAD bound to the helix that follows it in the ARM repeat of which it is a part (Figure 6A). This interaction is mediated by hydrophobic patches on both helices. LLPS by ASAD also involves hydrophobic interactions. The ASAD is a helical peptide with opposing patches: two hydrophobic patches (1 and 2, see Figure 6B) and a charged patch (Figure 6A,B). Therefore, APC may undergo LLPS as a result of hydrophobic collapse, to decrease the exposure of the hydrophobic patches of the ASAD to the solvent [28,51]. This raises the possibility that the hydrophobic interactions mediating the folding of the ASAD-containing ARM repeat are competing with the oligomerization of the ASAD in LLPS. The SLSSL repeat binds to the ASAD by interactions with both its charged patch and the first hydrophobic patch (Figure 2F and Figure 6B). This could lead to only the second hydrophobic patch mediating the interaction of the ASAD with helix 2 of the ARM repeat, leading to decreased affinity between these two helices, which in turn could cause their disassociation and lead to oligomerization (Figure 6D). Furthermore, these results can explain the contribution of the ASAD to the droplet formation of APC2 with Axin. APC2 is much shorter than APC, and lacks the N-terminal coils present in APC, which are predicted to partially obstruct the ARM region [52] (Figure 6C). Hence, the ASAD of APC2 is more exposed, enabling it to bind 20R3/5 and to form droplets.

Our results show that the ARM repeat can interact with different components of the 20R domain that are located more than 1000 residues removed toward the C-terminus. The binding of the ASAD to the 20Rs raises the possibility that APC can undergo oligomerization via these domains or intra-molecular interactions between them, resulting in partial folding of this largely intrinsically disordered protein. Such a folding event would bring the IDRs close to the folded N-terminal domain, the only well-structured region of APC. This in turn could regulate interactions of the N-terminal domain including cytoskeletal regulators (IQGAP, ASEF, and others). Mutations of APC lead to the expression of a truncated protein, lacking the 20R domain. Therefore, truncated APC cannot form ASAD-20R3/5 interactions, which would remove such potential regulation and explain why, for instance, N-terminal fragments expressed in tumor cells stimulate the ASEF protein more efficiently [53]. Further examining the effect of inhibiting ASAD-20R3/5 interactions in cells will reveal how these interactions affect the regulation of APC (Figure 6).

**Figure 7:**
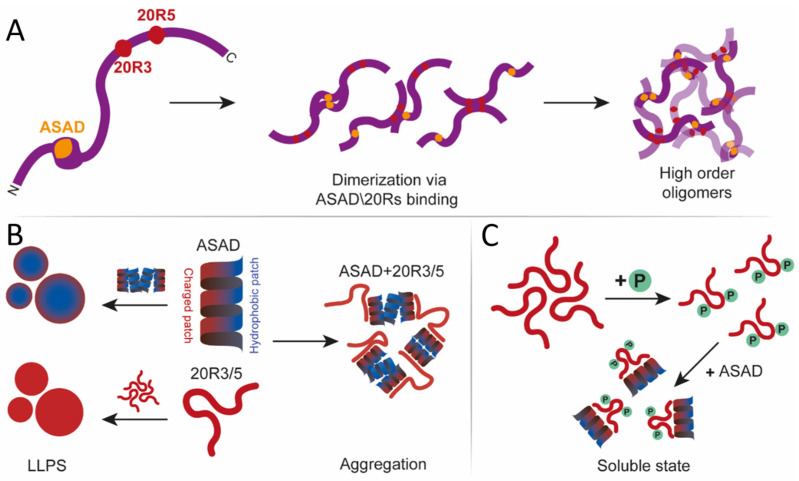
A scheme that describes oligomerization states of APC. (**A**) APC (purple) can dimerize and form higher-order structures via ASAD-ASAD (orange), 20R3-20R3, 20R5-20R5 interactions (red), and ASAD 20R3/5 interactions. (**B**) ASAD hydrophobic patches (blue) assemble droplets by hydrophobic interactions, while the charged patch (red) remains exposed to the polar solvent. 20R3/5 can form droplets by electrostatic interactions. While bound to each other, ASAD and 20Rs do not form droplets. (**C**) Phosphorylation of 20R3/5 prevent droplet formation.

In conclusion, oligomerization of APC can be mediated by interactions between three domains: the N-terminal domain, the ASAD, and the 20R3/5 to create numerous possibilities and combinations which can produce different states of APC to initiate different pathways (Figure 7A).

### 3.2. Droplet Formation of APC Is Mediated by Different Domains and Mechanisms

APC2 shares many of the well-conserved domains with human APC, including the ASAD, with some differences in the ASAD sequence [52] (Table 2). There are several substitutions of hydrophobic and charged residues in the ASAD of APC2 compared to human APC (R→L, E→N, W→H and W→Q). However, the substitutions tend to maintain the charged and hydrophobic nature. Consistently, wheel projections show that the opposing hydrophobic and charged patches of the ASAD are preserved. Therefore, the ASAD in both human APC and *drosophila* APC2 could form droplets via a hydrophobic patch, indicating that the function of ASAD in mediating and forming droplets is well-conserved between these two APC proteins.

### 3.3. The Role of the SLSSL Residues in Mediating Droplet Formation of the 20R Domain

The droplet formation of 20R5 occurs at a physiological and lower ionic strength, suggesting this LLPS is mediated by electrostatic interactions. On the other hand, LLPS of 20R3 was not affected by ionic strength to the same degree. These differences can be explained by differences in their sequences. First, the consensus SLSXL repeat of 20R3 has alanine on the 4th position, while 20R5 has a serine. This alone could affect the LLPS of both peptides. Furthermore, 20R5 is highly charged (33.3%) and polar (33.3%), and droplets formed by 20R5 are more likely to interact electrostatically. 20R3 is less charged (13.3%), and more polar (40%), which is consistent with the fact that its ability to form droplets was less affected by ionic strength. The 20R3 peptide we used formed droplets at identical PEG and ionic strength conditions to those reported for the longer 20R2-3 [40]. This suggests that the polarity and/or the relative charge of individual droplet-forming domains affect the details of the LLPS. Droplet formation by the SLSXL sequence is consistent with previous work showing that LLPS is driven by hydrophobic residues, linked by a polar spacer [50,54]. Consistently, 20R4 did not form droplets at any ionic strength, showing that not all the 20R repeats form droplets and emphasizing the importance of 20R3 and 20R5 for the process. 20R4 has an aspartic acid as the 4th residue of the SLSXL repeat, which is consistent with the idea that the 3rd and 4th serine within the SLSXL sequence are essential for droplet formation. Our results can explain the lack of droplet formation when the 20Rs and the ASAD were incubated together. The ASAD has both charged and hydrophobic patches. It undergoes LLPS by hydrophobic collapse, resulting in a hydrophobic core with a charged surface toward the polar solvent (Figure 7B). Binding of the SLSXL repeat to the ASAD via both its polar and hydrophobic patches may not allow the ASAD to bind itself via the hydrophobic patches that are necessary for its LLPS, thus preventing droplet formation when incubated together.

Finally, the SLSSL sequence is also found in the E-cadherin protein, another critical binding partner of β-catenin [32,41]. E-cadherin and APC compete for β-catenin, with E-cadherin having a higher affinity [32,41]. The SLSSL sequence in E-cadherin is important for its binding to β-catenin. This interaction depends on the phosphorylation of Ser3 in the SLSSL. This is thought to add rigidity to a flexible region, enabling the SLpSSL to fit into the binding groove of β-catenin [32,41]. Together with our findings that SLSSL is also necessary for binding 20R3/5 to the ASAD and that the phosphorylation of Ser 3 affects the LLPS of 20R3/5, this indicates that the SLSSL sequence is an important motif for mediating different protein-protein interactions.

### 3.4. Phosphorylation Regulates Droplet Formation by the 20R Domains of APC

Phosphorylation of the 20Rs increased their net charge and prevented droplet formation. This could be the result of the repulsion of the phosphate groups from each other, preventing the interactions required for LLPS, and/or increasing the solubility of the peptides in the polar solvent. 20R5 contains one CK1 phosphorylation site. We suggest that the mono-phosphopeptide does not contain sufficient charge to fully dissolve, or that the repulsion between the peptides is not strong enough to prevent droplet formation. This is supported by the fact that low ionic strength prevented droplet formation of 20R5 (Figure 7C). Our results show that phosphorylation can be an “on/off” switch for droplets formation.

In conclusion, our results show that the 20Rs are critical regulators of the oligomerization, interactions, and activity of APC. Their location in the middle of an IDR enables them to perform this important function and to regulate it by LLPS and phosphorylation, two properties of IDRs. In the case of APC, this combination of multiple oligomerization sites and control mechanisms enables the formation of different states that direct this highly important multifunctional protein to its different functions.

## 4. Materials and Methods

### 4.1. Peptide Synthesis, Labeling, Stapling, and Purification

The peptides were synthesized using a Liberty Blue Microwave-Assisted Peptide Synthesizer (CEM) as described previously [55]. Briefly, standard Fmoc chemistry with Oxyma (Luxembourg bio technologies LTD, Nes Ziona, Israel)/DIC (Chem-impex INT’L, Wood Dale, IL, USA) as coupling reagents was used. The peptide concentrations were measured using UV spectroscopy after the addition of Trp to the N-termini of the peptides. For the fluorescence anisotropy binding studies, the peptides were labeled with 5(6)-carboxyfluorescein (Sigma-aldrich, St. Louis, MO, USA) or rhodamine b (Alfa-aesar, Lancashire, UK) at their N′ termini [56], and cleaved from the resin as described [57]. The peptides were purified on a MERCK Hitachi HPLC using a reverse-phase C18 preparative column with a gradient of ACN/TDW. The identity and purity of the peptides was verified by ESI mass spectrometry and analytical HPLC. Phosphorylated peptides synthesis was carried out using the protocols described before [58].

### 4.2. Peptide Array

INTAVIS Bioanalytical Instruments AG, Köln, Germany, synthesized the CelluSpots^TM^ peptide micro-arrays [55]. The peptides were attached to a cellulose membrane through an amide bond at their C termini and acetylated at their N-termini. For screening the binding ASAD to APC, the ASAD was synthesized with a His tag (6xHis on the N′ terminus of the peptide). The array was first washed for four hours at room temperature with 50 mM Tris-HCl (Fisher scientific, Geel, Belgium) pH = 7.4 at an ionic strength of 150 mM adjusted by NaCl (Biolab-Biology Ltd., Jerusalem, Israel), 0.05% Tween20 and 2.5% (*w/v*) skimmed milk for blocking unspecific binding. Then 1 mM of the peptide dissolved in buffer A, including 4% (*w/v*) skimmed milk (Becton, Dickinson and Co., Franklin Lakes, New Jersey), was incubated overnight with the array at room temperature with shaking. After three washes with TBST, the array was incubated at room temperature for one hour with an anti-His HRP-conjugated antibody dissolved in buffer A (Biological Industries, Beit Ha’emek, Israel), followed by three additional washes of the array with TBST. Immunodetection was performed using chemiluminescence (ECL reagents).

### 4.3. DIC Microscopy

30 µM peptides were incubated and vortexed for 3–5 min in 25% PEG400 (Sigma-Aldrich catalog number: 25322-68-3) and 10 mM phosphate buffer in various ionic strength conditions, adjusted by 1M NaCl solution (LLPS buffer) [59]. Then 10–20 µL of the mixture was pipetted onto a microscopy glass slide and imaged. The phase separation behavior of peptides was studied using a Zeiss Axio Scope A1 microscope (Carl Zeiss Pte Ltd., Oberkochen, Germany) in the reflection mode, with differential interference contrast (DIC) filters. Images were taken with an AxioCam ICc 3 camera under the control of Zen software.

### 4.4. Circular Dichroism

The CD spectra of all peptides were recorded in the same manner as previously [55] with a J-810 spectropolarimeter (JASCO Inc., Easton, PA, USA) and its supplied Spectra Manager software, using a Peltier thermostat, in a 0.1 cm quartz cuvette for far-UV CD spectroscopy, in the range of 197 nm to 260 nm.

### 4.5. Contact Angle Measurements

The contact angle between ~1 µL of the ASAD in LLPS buffer solution and a glass slip covered with parafilm (Bemis, Sparks, MD, USA) was measured at the interface between the glass and line tangent to the droplet using a Theta Lite optical tensiometer (Attension, Espoo, Finland) and Attension Theta software. Each result is composed of an average of 3 repeats with 100 measurements.

## Figures and Tables

**Table 1:** The 20R peptides and their phosphorylation patterns.

Peptide	Sequence
20R3	GFSCSSSLSALSLDE
20R3CK1(pS1504,1507)	GFSCSpSSLpSALSLDE
20R3GSK3β(pS1501,1505)	GFpSCSSpSLSALSLDE
20R5	CFSRNDSLSSLDFDD
20R5CK1(pS1863)	CFSRNDSLpSSLDFDD
20R5GSK3β(pS1857,1861)	CFpSRNDpSLSSLDFDD
20R4	NFSTATSLSDLTIES

**Table 2:** A comparison between Human APC and *Drosophila* APC2 *.

Peptide	Sequence	Wheel Projection
Human APC	LHLLEQI**RA**YC**ETCW**E**WQ**E**A**	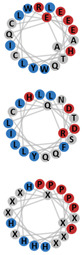
*Drosophila* APC2	LRLLDQI**LD**YC**NFLH**T**QL**Q**S**	
Consensus	HPHHPPH – – P – – – – – P – – P –	

* Relevant differences between the ASAD in human APC and APC2 are labeled in bold. The consensus hydrophobicity pattern is also shown (Polar/Charged = P, Hydrophobic = H). On the right, wheel projections reveal conservation of separate charged and hydrophobic patches. All projections were created using pepwheel (https://www.bioinformatics.nl/cgi-bin/emboss/pepwheel, accessed on 19 December 2019).

## Data Availability

Data is available from the corresponding authors upon reasonable request.

## Supplementary Materials

The following supporting information can be downloaded at: https://www.mdpi.com/article/10.3390/ijms24076478/s1, Table S1: Peptides derived from APC included in the peptide array*; Figure S1: The effect of phosphorylation on droplet formation by APC derived peptides (gray scale copy of Figure 4): (A) ASAD labeled with fluorescein and 20R3 labeled with rhodamine b located to the same clusters (B) Phosphorylation of 20R3 prevented co-aggregation with ASAD. (C) ASAD labeled with fluorescein and 20R5 labeled with rhodamine b located to the same clusters (D) Phosphorylation of 20R5 prevented co-aggregating with ASAD. All scale bars = 20 μm.
