# Supplementary material for: The Oligomerization Domains of the APC Protein Mediate Liquid-Liquid Phase Separation That Is Phosphorylation Controlled
Source: Int J Mol Sci. 2023 Mar 30;24(7):6478. doi: 10.3390/ijms24076478 (PMC10095272; doi:10.3390/ijms24076478)
Supplement: Supplementary file 1 [file ijms-24-06478-s001.zip › ijms-2253433-supplementary.pdf]

Supplementary Materials

The Oligomerization Domains of the APC Protein Mediate Liquid-Liquid Phase Separation That Is Phosphorylation Controlled

Shachar G. Bressler [1], Amit Mitrany [1], Alon Wenger [1], Inke Näthke [2,*] and Assaf Friedler [1,*]

[1] The Institute of Chemistry, The Hebrew University of Jerusalem, Edmond J. Safra Campus, Givat Ram, Jerusalem 91904, Israel
[2] Division of Molecular Cell and Developmental Biology, University of Dundee, Dundee DD1 5AA, Scotland, UK
* Correspondence: i.s.nathke@dundee.ac.uk (I.N.); assaf.friedler@mail.huji.ac.il (A.F.)

Table S1. Peptides derived from APC included in the peptide array*.

Location on the array	sequence	Residues numbers
A 1	M-A-A-A-S-Y-D-Q-L-L-K-Q-V-E-A	APC 1-15
A 2	K-Q-V-E-A-L-K-M-E-N-S-N-L-R-Q	APC 11-25
A 3	S-N-L-R-Q-E-L-E-D-N-S-N-H-L-T	APC 21-35
A 4	S-N-H-L-T-K-L-E-T-E-A-S-N-M-K	APC 31-45
A 5	A-S-N-M-K-E-V-L-K-Q-L-Q-G-S-I	APC 41-55
A 6	L-Q-G-S-I-E-D-E-A-M-A-S-S-G-Q	APC 51-65
A 7	A-S-S-G-Q-I-D-L-L-E-R-L-K-E-L	APC 61-75
A 8	R-L-K-E-L-N-L-D-S-S-N-F-P-G-V	APC 71-85
A 9	N-F-P-G-V-K-L-R-S-K-M-S-L-R-S	APC 81-95
A10	M-S-L-R-S-Y-G-S-R-E-G-S-V-S-S	APC 91-105
A11	G-S-V-S-S-R-S-G-E-C-S-P-V-P-M	APC 101-115
A12	S-P-V-P-M-G-S-F-P-R-R-G-F-V-N	APC 111-125
A13	R-G-F-V-N-G-S-R-E-S-T-G-Y-L-E	APC 121-135
A14	T-G-Y-L-E-E-L-E-K-E-R-S-L-L-L	APC 131-145
A15	R-S-L-L-L-A-D-L-D-K-E-E-K-E-K	APC 141-155
A16	E-E-K-E-K-D-W-Y-Y-A-Q-L-Q-N-L	APC 151-165
A17	Q-L-Q-N-L-T-K-R-I-D-S-L-P-L-T	APC 161-175
A18	S-L-P-L-T-E-N-F-S-L-Q-T-D-M-T	APC 171-185
A19	Q-T-D-M-T-R-R-Q-L-E-Y-E-A-R-Q	APC 181-195
A20	Y-E-A-R-Q-I-R-V-A-M-E-E-Q-L-G	APC 191-205
A21	E-E-Q-L-G-T-C-Q-D-M-E-K-R-A-Q	APC 201-215
A22	E-K-R-A-Q-R-R-I-A-R-I-Q-Q-I-E	APC 211-225
A23	I-Q-Q-I-E-K-D-I-L-R-I-R-Q-L-L	APC 221-235
A24	I-R-Q-L-L-Q-S-Q-A-T-E-A-E-R-S	APC 231-245
B 1	E-A-E-R-S-S-Q-N-K-H-E-T-G-S-H	APC 241-255
B 2	E-T-G-S-H-D-A-E-R-Q-N-E-G-Q-G	APC 251-265
B 3	N-E-G-Q-G-V-G-E-I-N-M-A-T-S-G	APC 261-275
B 4	M-A-T-S-G-N-G-Q-G-S-T-T-R-M-D	APC 271-285
B 5	T-T-R-M-D-H-E-T-A-S-V-L-S-S-S	APC 281-295
B 6	V-L-S-S-S-S-T-H-S-A-P-R-R-L-T	APC 291-305
B 7	P-R-R-L-T-S-H-L-G-T-K-V-E-M-V	APC 301-315
B 8	K-V-E-M-V-Y-S-L-L-S-M-L-G-T-H	APC 311-325
B 9	M-L-G-T-H-D-K-D-D-M-S-R-T-L-L	APC 321-335
B10	S-R-T-L-L-A-M-S-S-S-Q-D-S-C-I	APC 331-345
B11	Q-D-S-C-I-S-M-R-Q-S-G-C-L-P-L	APC 341-355
B12	G-C-L-P-L-L-I-Q-L-L-H-G-N-D-K	APC 351-365
B13	H-G-N-D-K-D-S-V-L-L-G-N-S-R-G	APC 361-375

B14	G-N-S-R-G-S-K-E-A-R-A-R-A-S-A	APC 371-385
B15	A-R-A-S-A-A-L-H-N-I-I-H-S-Q-P	APC 381-395
B16	I-H-S-Q-P-D-D-K-R-G-R-R-E-I-R	APC 391-405
**B17**	**R-R-E-I-R-V-L-H-L-L-E-Q-I-R-A**	**APC 401-415**
B18	E-Q-I-R-A-Y-C-E-T-C-W-E-W-Q-E	APC 411-425
B19	W-E-W-Q-E-A-H-E-P-G-M-D-Q-D-K	APC 421-435
B20	M-D-Q-D-K-N-P-M-P-A-P-V-E-H-Q	APC 431-445
B21	V-E-H-Q-I-C-P-A-V-C-V-L-M-K-L	APC 442-456
B22	C-V-L-M-K-L-S-F-D-E-E-H-R-H-A	APC 451-465
B23	E-E-H-R-H-A-M-N-E-L-G-G-L-Q-A	APC 460-474
**B24**	**G-L-Q-A-I-A-E-L-L-Q-V-D-C-E-M-Y**	**APC 471-486**
C 1	V-D-C-E-M-Y-G-L-T-N-D-H-Y-S-I	APC 481-495
C 2	D-H-Y-S-I-T-L-R-R-Y-A-G-M-A-L	APC 491-505
C 3	A-G-M-A-L-T-N-L-T-F-G-D-V-A-N	APC 501-515
C 4	G-D-V-A-N-K-A-T-L-C-S-M-K-G-C	APC 511-525
C 5	S-M-K-G-C-M-R-A-L-V-A-Q-L-K-S	APC 521-535
C 6	A-Q-L-K-S-E-S-E-D-L-Q-Q-V-I-A	APC 531-545
C 7	Q-Q-V-I-A-S-V-L-R-N-L-S-W-R-A	APC 541-555
C 8	L-S-W-R-A-D-V-N-S-K-K-T-L-R-E	APC 551-565
C 9	K-T-L-R-E-V-G-S-V-K-A-L-M-E-C	APC 561-575
C10	A-L-M-E-C-A-L-E-V-K-K-E-S-T-L	APC 571-585
C11	K-E-S-T-L-K-S-V-L-S-A-L-W-N-L	APC 581-595
C12	A-L-W-N-L-S-A-H-C-T-E-N-K-A-D	APC 591-605
C13	T-E-N-K-A-D-I-C-A-V-D-G-A-L-A	APC 600-614
C14	G-A-L-A-F-L-V-G-T-L-T-Y-R-S-Q	APC 611-625
C15	T-Y-R-S-Q-T-N-T-L-A-I-I-E-S-G	APC 621-635
C16	I-I-E-S-G-G-G-I-L-R-N-V-S-S-L	APC 631-645
C17	N-V-S-S-L-I-A-T-N-E-D-H-R-Q-I	APC 641-655
C18	E-D-H-R-Q-I-L-R-E-N-N-C-L-Q-T	APC 650-664
C19	C-L-Q-T-L-L-Q-H-L-K-S-H-S-L-T	APC 661-675
C20	S-H-S-L-T-I-V-S-N-A-C-G-T-L-W	APC 671-685
C21	C-G-T-L-W-N-L-S-A-R-N-P-K-D-Q	APC 681-695
C22	N-P-K-D-Q-E-A-L-W-D-M-G-A-V-S	APC 691-705
C23	M-G-A-V-S-M-L-K-N-L-I-H-S-K-H	APC 701-715
C24	I-H-S-K-H-K-M-I-A-M-G-S-A-A-A	APC 711-725
D 1	G-S-A-A-A-L-R-N-L-M-A-N-R-P-A	APC 721-735
D 2	A-N-R-P-A-K-Y-K-D-A-N-I-M-S-P	APC 731-745
D 3	N-I-M-S-P-G-S-S-L-P-S-L-H-V-R	APC 741-755
D 4	S-L-H-V-R-K-Q-K-A-L-E-A-E-L-D	APC 751-765
D 5	E-A-E-L-D-A-Q-H-L-S-E-T-F-D-N	APC 761-775
D 6	E-T-F-D-N-I-D-N-L-S-P-K-A-S-H	APC 771-785
D 7	P-K-A-S-H-R-S-K-Q-R-H-K-Q-S-L	APC 781-795
D 8	H-K-Q-S-L-Y-G-D-Y-V-F-D-T-N-R	APC 791-805
D 9	F-D-T-N-R-H-D-D-N-R-S-D-N-F-N	APC 801-815
D10	S-D-N-F-N-T-G-N-M-T-V-L-S-P-Y	APC 811-825
D11	V-L-S-P-Y-L-N-T-T-V-L-P-S-S-S	APC 821-835
D12	L-P-S-S-S-S-S-R-G-S-L-D-S-S-R	APC 831-845
D13	L-D-S-S-R-S-E-K-D-R-S-L-E-R-E	APC 841-855
D14	S-L-E-R-E-R-G-I-G-L-G-N-Y-H-P	APC 851-865
D15	G-N-Y-H-P-A-T-E-N-P-G-T-S-S-K	APC 861-875
D16	G-T-S-S-K-R-G-L-Q-I-S-T-T-A-A	APC 871-885

D17	S-T-T-A-A-Q-I-A-K-V-M-E-E-V-S	APC 881-895
D18	M-E-E-V-S-A-I-H-T-S-Q-E-D-R-S	APC 891-905
D19	Q-E-D-R-S-S-G-S-T-T-E-L-H-C-V	APC 901-915
D20	E-L-H-C-V-T-D-E-R-N-A-L-R-R-S	APC 911-925
D21	A-L-R-R-S-S-A-A-H-T-H-S-N-T-Y	APC 921-935
D22	H-S-N-T-Y-N-F-T-K-S-E-N-S-N-R	APC 931-945
D23	E-N-S-N-R-T-C-S-M-P-Y-A-K-L-E	APC 941-955
D24	Y-A-K-L-E-Y-K-R-S-S-N-D-S-L-N	APC 951-965
E 1	N-D-S-L-N-S-V-S-S-S-D-G-Y-G-K	APC 961-975
E 2	D-G-Y-G-K-R-G-Q-M-K-P-S-I-E-S	APC 971-985
E 3	P-S-I-E-S-Y-S-E-D-D-E-S-K-F-C	APC 981-995
E 4	E-S-K-F-C-S-Y-G-Q-Y-P-A-D-L-A	APC 991-1005
E 5	P-A-D-L-A-H-K-I-H-S-A-N-H-M-D	APC 1001-1015
E 6	A-N-H-M-D-D-N-D-G-E-L-D-T-P-I	APC 1011-1025
E 7	L-D-T-P-I-N-Y-S-L-K-Y-S-D-E-Q	APC 1021-1035
E 8	Y-S-D-E-Q-L-N-S-G-R-Q-S-P-S-Q	APC 1031-1045
E 9	Q-S-P-S-Q-N-E-R-W-A-R-P-K-H-I	APC 1041-1055
E10	R-P-K-H-I-I-E-D-E-I-K-Q-S-E-Q	APC 1051-1065
E11	K-Q-S-E-Q-R-Q-S-R-N-Q-S-T-T-Y	APC 1061-1075
E12	Q-S-T-T-Y-P-V-Y-T-E-S-T-D-D-K	APC 1071-1085
E13	S-T-D-D-K-H-L-K-F-Q-P-H-F-G-Q	APC 1081-1095
E14	P-H-F-G-Q-Q-E-C-V-S-P-Y-R-S-R	APC 1091-1105
E15	P-Y-R-S-R-G-A-N-G-S-E-T-N-R-V	APC 1101-1115
E16	E-T-N-R-V-G-S-N-H-G-I-N-Q-N-V	APC 1111-1125
E17	I-N-Q-N-V-S-Q-S-L-C-Q-E-D-D-Y	APC 1121-1135
E18	Q-E-D-D-Y-E-D-D-K-P-T-N-Y-S-E	APC 1131-1145
E19	T-N-Y-S-E-R-Y-S-E-E-E-Q-H-E-E	APC 1141-1155
E20	E-Q-H-E-E-E-E-R-P-T-N-Y-S-I-K	APC 1151-1165
E21	N-Y-S-I-K-Y-N-E-E-K-R-H-V-D-Q	APC 1161-1175
E22	R-H-V-D-Q-P-I-D-Y-S-L-K-Y-A-T	APC 1171-1185
E23	L-K-Y-A-T-D-I-P-S-S-Q-K-Q-S-F	APC 1181-1195
E24	Q-K-Q-S-F-S-F-S-K-S-S-S-G-Q-S	APC 1191-1205
F 1	S-S-G-Q-S-S-K-T-E-H-M-S-S-S-S	APC 1201-1215
F 2	M-S-S-S-S-E-N-T-S-T-P-S-S-N-A	APC 1211-1225
F 3	P-S-S-N-A-K-R-Q-N-Q-L-H-P-S-S	APC 1221-1235
F 4	L-H-P-S-S-A-Q-S-R-S-G-Q-P-Q-K	APC 1231-1245
F 5	G-Q-P-Q-K-A-A-T-C-K-V-S-S-I-N	APC 1241-1255
F 6	V-S-S-I-N-Q-E-T-I-Q-T-Y-C-V-E	APC 1251-1265
F 7	T-Y-C-V-E-D-T-P-I-C-F-S-R-C-S	APC 1261-1275
F 8	F-S-R-C-S-S-L-S-S-L-S-S-A-E-D	APC 1271-1285
F 9	S-S-A-E-D-E-I-G-C-N-Q-T-T-Q-E	APC 1281-1295
F10	Q-T-T-Q-E-A-D-S-A-N-T-L-Q-I-A	APC 1291-1305
F11	T-L-Q-I-A-E-I-K-E-K-I-G-T-R-S	APC 1301-1315
F12	I-G-T-R-S-A-E-D-P-V-S-E-V-P-A	APC 1311-1325
F13	S-E-V-P-A-V-S-Q-H-P-R-T-K-S-S	APC 1321-1335
F14	R-T-K-S-S-R-L-Q-G-S-S-L-S-S-E	APC 1331-1345
F15	S-L-S-S-E-S-A-R-H-K-A-V-E-F-S	APC 1341-1355
F16	A-V-E-F-S-S-G-A-K-S-P-S-K-S-G	APC 1351-1365
F17	P-S-K-S-G-A-Q-T-P-K-S-P-P-E-H	APC 1361-1375
F18	S-P-P-E-H-Y-V-Q-E-T-P-L-M-F-S	APC 1371-1385
F19	P-L-M-F-S-R-C-T-S-V-S-S-L-D-S	APC 1381-1395

F20	S-S-L-D-S-F-E-S-R-S-I-A-S-S-V	APC 1391-1405
F21	I-A-S-S-V-Q-S-E-P-C-S-G-M-V-S	APC 1401-1415
F22	S-G-M-V-S-G-I-I-S-P-S-D-L-P-D	APC 1411-1425
F23	S-D-L-P-D-S-P-G-Q-T-M-P-P-S-R	APC 1421-1435
F24	M-P-P-S-R-S-K-T-P-P-P-P-P-Q-T	APC 1431-1445
G 1	P-P-P-Q-T-A-Q-T-K-R-E-V-P-K-N	APC 1441-1455
G 2	E-V-P-K-N-K-A-P-T-A-E-K-R-E-S	APC 1451-1465
G 3	E-K-R-E-S-G-P-K-Q-A-A-V-N-A-A	APC 1461-1475
G 4	A-A-V-N-A-A-V-Q-R-V-Q-V-L-P-D	APC 1470-1484
G 5	V-L-P-D-A-D-T-L-L-H-F-A-T-E-S	APC 1481-1495
G 6	F-A-T-E-S-T-P-D-G-F-S-C-S-S-S	APC 1491-1505
**G 7**	**S-C-S-S-S-L-S-A-L-S-L-D-E-P-F**	**APC 1501-1515**
G 8	L-D-E-P-F-I-Q-K-D-V-E-L-R-I-M	APC 1511-1525
G 9	E-L-R-I-M-P-P-V-Q-E-N-D-N-G-N	APC 1521-1535
G10	N-D-N-G-N-E-T-E-S-E-Q-P-K-E-S	APC 1531-1545
G11	Q-P-K-E-S-N-E-N-Q-E-K-E-A-E-K	APC 1541-1555
G12	K-E-A-E-K-T-I-D-S-E-K-D-L-L-D	APC 1551-1565
G13	K-D-L-L-D-D-S-D-D-D-D-I-E-I-L	APC 1561-1575
G14	D-I-E-I-L-E-E-C-I-I-S-A-M-P-T	APC 1571-1585
G15	S-A-M-P-T-K-S-S-R-K-A-K-K-P-A	APC 1581-1595
G16	A-K-K-P-A-Q-T-A-S-K-L-P-P-P-V	APC 1591-1605
G17	L-P-P-P-V-A-R-K-P-S-Q-L-P-V-Y	APC 1601-1615
G18	Q-L-P-V-Y-K-L-L-P-S-Q-N-R-L-Q	APC 1611-1625
G19	Q-N-R-L-Q-P-Q-K-H-V-S-F-T-P-G	APC 1621-1635
G20	S-F-T-P-G-D-D-M-P-R-V-Y-C-V-E	APC 1631-1645
G21	V-Y-C-V-E-G-T-P-I-N-F-S-T-A-T	APC 1641-1655
G22	F-S-T-A-T-S-L-S-D-L-T-I-E-S-P	APC 1651-1665
G23	T-I-E-S-P-P-N-E-L-A-A-G-E-G-V	APC 1661-1675
G24	A-G-E-G-V-R-G-G-A-Q-S-G-E-F-E	APC 1671-1685
H 1	S-G-E-F-E-K-R-D-T-I-P-T-E-G-R	APC 1681-1695
H 2	P-T-E-G-R-S-T-D-E-A-Q-G-G-K-T	APC 1691-1705
H 3	Q-G-G-K-T-S-S-V-T-I-P-E-L-D-D	APC 1701-1715
H 4	P-E-L-D-D-N-K-A-E-E-G-D-I-L-A	APC 1711-1725
H 5	G-D-I-L-A-E-C-I-N-S-A-M-P-K-G	APC 1721-1735
H 6	A-M-P-K-G-K-S-H-K-P-F-R-V-K-K	APC 1731-1745
H 7	F-R-V-K-K-I-M-D-Q-V-Q-Q-A-S-A	APC 1741-1755
H 8	Q-Q-A-S-A-S-S-S-A-P-N-K-N-Q-L	APC 1751-1765
H 9	N-K-N-Q-L-D-G-K-K-K-K-P-T-S-P	APC 1761-1775
H10	K-P-T-S-P-V-K-P-I-P-Q-N-T-E-Y	APC 1771-1785
H11	Q-N-T-E-Y-R-T-R-V-R-K-N-A-D-S	APC 1781-1795
H12	K-N-A-D-S-K-N-N-L-N-A-E-R-V-F	APC 1791-1805
H13	A-E-R-V-F-S-D-N-K-D-S-K-K-Q-N	APC 1801-1815
H14	S-K-K-Q-N-L-K-N-N-S-K-V-F-N-D	APC 1811-1825
H15	K-V-F-N-D-K-L-P-N-N-E-D-R-V-R	APC 1821-1835
H16	E-D-R-V-R-G-S-F-A-F-D-S-P-H-H	APC 1831-1845
H17	D-S-P-H-H-Y-T-P-I-E-G-T-P-Y-C	APC 1841-1855
H18	G-T-P-Y-C-F-S-R-N-D-S-L-S-S-L	APC 1851-1865
**H19**	**S-L-S-S-L-D-F-D-D-D-D-V-D-L-S**	**APC 1861-1875**
H20	D-V-D-L-S-R-E-K-A-E-L-R-K-A-K	APC 1871-1885
H21	L-R-K-A-K-E-N-K-E-S-E-A-K-V-T	APC 1881-1895
H22	E-A-K-V-T-S-H-T-E-L-T-S-N-Q-Q	APC 1891-1905

H23	T-S-N-Q-Q-S-A-N-K-T-Q-A-I-A-K	APC 1901-1915
H24	Q-A-I-A-K-Q-P-I-N-R-G-Q-P-K-P	APC 1911-1925
I 1	G-Q-P-K-P-I-L-Q-K-Q-S-T-F-P-Q	APC 1921-1935
I 2	S-T-F-P-Q-S-S-K-D-I-P-D-R-G-A	APC 1931-1945
I 3	P-D-R-G-A-A-T-D-E-K-L-Q-N-F-A	APC 1941-1955
I 4	L-Q-N-F-A-I-E-N-T-P-V-C-F-S-H	APC 1951-1965
I 5	V-C-F-S-H-N-S-S-L-S-S-L-S-D-I	APC 1961-1975
I 6	S-L-S-D-I-D-Q-E-N-N-N-K-E-N-E	APC 1971-1985
I 7	N-K-E-N-E-P-I-K-E-T-E-P-P-D-S	APC 1981-1995
I 8	E-P-P-D-S-Q-G-E-P-S-K-P-Q-A-S	APC 1991-2005
I 9	K-P-Q-A-S-G-Y-A-P-K-S-F-H-V-E	APC 2001-2015
I10	S-F-H-V-E-D-T-P-V-C-F-S-R-N-S	APC 2011-2025
I11	F-S-R-N-S-S-L-S-S-L-S-I-D-S-E	APC 2021-2035
I12	S-I-D-S-E-D-D-L-L-Q-E-C-I-S-S	APC 2031-2045
I13	E-C-I-S-S-A-M-P-K-K-K-K-P-S-R	APC 2041-2055
I14	K-K-P-S-R-L-K-G-D-N-E-K-H-S-P	APC 2051-2065
I15	E-K-H-S-P-R-N-M-G-G-I-L-G-E-D	APC 2061-2075
I16	I-L-G-E-D-L-T-L-D-L-K-D-I-Q-R	APC 2071-2085
I17	K-D-I-Q-R-P-D-S-E-H-G-L-S-P-D	APC 2081-2095
I18	G-L-S-P-D-S-E-N-F-D-W-K-A-I-Q	APC 2091-2105
I19	W-K-A-I-Q-E-G-A-N-S-I-V-S-S-L	APC 2101-2115
I20	I-V-S-S-L-H-Q-A-A-A-A-A-C-L-S	APC 2111-2125
I21	A-A-C-L-S-R-Q-A-S-S-D-S-D-S-I	APC 2121-2135
I22	D-S-D-S-I-L-S-L-K-S-G-I-S-L-G	APC 2131-2145
I23	G-I-S-L-G-S-P-F-H-L-T-P-D-Q-E	APC 2141-2155
I24	T-P-D-Q-E-E-K-P-F-T-S-N-K-G-P	APC 2151-2165
J 1	S-N-K-G-P-R-I-L-K-P-G-E-K-S-T	APC 2161-2175
J 2	G-E-K-S-T-L-E-T-K-K-I-E-S-E-S	APC 2171-2185
J 3	I-E-S-E-S-K-G-I-K-G-G-K-K-V-Y	APC 2181-2195
J 4	G-K-K-V-Y-K-S-L-I-T-G-K-V-R-S	APC 2191-2205
J 5	G-K-V-R-S-N-S-E-I-S-G-Q-M-K-Q	APC 2201-2215
J 6	G-Q-M-K-Q-P-L-Q-A-N-M-P-S-I-S	APC 2211-2225
J 7	M-P-S-I-S-R-G-R-T-M-I-H-I-P-G	APC 2221-2235
J 8	I-H-I-P-G-V-R-N-S-S-S-S-T-S-P	APC 2231-2245
J 9	S-S-T-S-P-V-S-K-K-G-P-P-L-K-T	APC 2241-2255
J10	P-P-L-K-T-P-A-S-K-S-P-S-E-G-Q	APC 2251-2265
J11	P-S-E-G-Q-T-A-T-T-S-P-R-G-A-K	APC 2261-2275
J12	P-R-G-A-K-P-S-V-K-S-E-L-S-P-V	APC 2271-2285
J13	E-L-S-P-V-A-R-Q-T-S-Q-I-G-G-S	APC 2281-2295
J14	Q-I-G-G-S-S-K-A-P-S-R-S-G-S-R	APC 2291-2305
J15	R-S-G-S-R-D-S-T-P-S-R-P-A-Q-Q	APC 2301-2315
J16	R-P-A-Q-Q-P-L-S-R-P-I-Q-S-P-G	APC 2311-2325
J17	I-Q-S-P-G-R-N-S-I-S-P-G-R-N-G	APC 2321-2335
J18	P-G-R-N-G-I-S-P-P-N-K-L-S-Q-L	APC 2331-2345
J19	K-L-S-Q-L-P-R-T-S-S-P-S-T-A-S	APC 2341-2355
J20	P-S-T-A-S-T-K-S-S-G-S-G-K-M-S	APC 2351-2365
J21	S-G-K-M-S-Y-T-S-P-G-R-Q-M-S-Q	APC 2361-2375
J22	R-Q-M-S-Q-Q-N-L-T-K-Q-T-G-L-S	APC 2371-2385
J23	Q-T-G-L-S-K-N-A-S-S-I-P-R-S-E	APC 2381-2395
J24	I-P-R-S-E-S-A-S-K-G-L-N-Q-M-N	APC 2391-2405
K 1	L-N-Q-M-N-N-G-N-G-A-N-K-K-V-E	APC 2401-2415

K 2	N-K-K-V-E-L-S-R-M-S-S-T-K-S-S	APC 2411-2425
K 3	S-T-K-S-S-G-S-E-S-D-R-S-E-R-P	APC 2421-2435
K 4	R-S-E-R-P-V-L-V-R-Q-S-T-F-I-K	APC 2431-2445
K 5	S-T-F-I-K-E-A-P-S-P-T-L-R-R-K	APC 2441-2455
K 6	T-L-R-R-K-L-E-E-S-A-S-F-E-S-L	APC 2451-2465
K 7	S-F-E-S-L-S-P-S-S-R-P-A-S-P-T	APC 2461-2475
K 8	P-A-S-P-T-R-S-Q-A-Q-T-P-V-L-S	APC 2471-2485
K 9	T-P-V-L-S-P-S-L-P-D-M-S-L-S-T	APC 2481-2495
K10	M-S-L-S-T-H-S-S-V-Q-A-G-G-W-R	APC 2491-2505
K11	A-G-G-W-R-K-L-P-P-N-L-S-P-T-I	APC 2501-2515
K12	L-S-P-T-I-E-Y-N-D-G-R-P-A-K-R	APC 2511-2525
K13	R-P-A-K-R-H-D-I-A-R-S-H-S-E-S	APC 2521-2535
K14	S-H-S-E-S-P-S-R-L-P-I-N-R-S-G	APC 2531-2545
K15	I-N-R-S-G-T-W-K-R-E-H-S-K-H-S	APC 2541-2555
K16	H-S-K-H-S-S-S-L-P-R-V-S-T-W-R	APC 2551-2565
K17	V-S-T-W-R-R-T-G-S-S-S-S-I-L-S	APC 2561-2575
K18	S-S-I-L-S-A-S-S-E-S-S-E-K-A-K	APC 2571-2585
K19	S-E-K-A-K-S-E-D-E-K-H-V-N-S-I	APC 2581-2595
K20	H-V-N-S-I-S-G-T-K-Q-S-K-E-N-Q	APC 2591-2605
K21	S-K-E-N-Q-V-S-A-K-G-T-W-R-K-I	APC 2601-2615
K22	T-W-R-K-I-K-E-N-E-F-S-P-T-N-S	APC 2611-2625
K23	S-P-T-N-S-T-S-Q-T-V-S-S-G-A-T	APC 2621-2635
K24	S-S-G-A-T-N-G-A-E-S-K-T-L-I-Y	APC 2631-2645
L 1	K-T-L-I-Y-Q-M-A-P-A-V-S-K-T-E	APC 2641-2655
L 2	V-S-K-T-E-D-V-W-V-R-I-E-D-C-P	APC 2651-2665
L 3	I-E-D-C-P-I-N-N-P-R-S-G-R-S-P	APC 2661-2675
L 4	S-G-R-S-P-T-G-N-T-P-P-V-I-D-S	APC 2671-2685
L 5	P-V-I-D-S-V-S-E-K-A-N-P-N-I-K	APC 2681-2695
L 6	N-P-N-I-K-D-S-K-D-N-Q-A-K-Q-N	APC 2691-2705
L 7	Q-A-K-Q-N-V-G-N-G-S-V-P-M-R-T	APC 2701-2715
L 8	V-P-M-R-T-V-G-L-E-N-R-L-N-S-F	APC 2711-2725
L 9	R-L-N-S-F-I-Q-V-D-A-P-D-Q-K-G	APC 2721-2735
L10	P-D-Q-K-G-T-E-I-K-P-G-Q-N-N-P	APC 2731-2745
L11	G-Q-N-N-P-V-P-V-S-E-T-N-E-S-S	APC 2741-2755
L12	T-N-E-S-S-I-V-E-R-T-P-F-S-S-S	APC 2751-2765
L13	P-F-S-S-S-S-S-S-K-H-S-S-P-S-G	APC 2761-2775
L14	S-S-P-S-G-T-V-A-A-R-V-T-P-F-N	APC 2771-2785
L15	V-T-P-F-N-Y-N-P-S-P-R-K-S-S-A	APC 2781-2795
L16	R-K-S-S-A-D-S-T-S-A-R-P-S-Q-I	APC 2791-2805
L17	R-P-S-Q-I-P-T-P-V-N-N-N-T-K-K	APC 2801-2815
L18	N-N-T-K-K-R-D-S-K-T-D-S-T-E-S	APC 2811-2825
L19	D-S-T-E-S-S-G-T-Q-S-P-K-R-H-S	APC 2821-2835
L20	P-K-R-H-S-G-S-Y-L-V-T-S-V	APC 2831-2843
L21	Y-D-Q-L-L-K-Q-V-E-A-L-K-M-E-N-S-N-L-R-Q	APC 6 – 25
L22	L-K-M-E-N-S-N-L-R-Q-E-L-E-D-N-S-N-H-L-T	APC 16 – 35
L23	E-L-E-D-N-S-N-H-L-T-K-L-E-T-E-A-S-N-M-K	APC 26 – 45
L24	K-L-E-T-E-A-S-N-M-K-E-V-L-K-Q-L-Q-G	APC 36 – 53
M 1	G-Y-L-E-E-L-E-K-E-R-S-L-L-L-A-D-L-D-K-E	APC 132 - 151
M 2	S-L-L-L-A-D-L-D-K-E-E-K-E-K-D-W-Y-Y-A-Q	APC 142 - 161
M 3	K-E-E-K-E-K-D-W-Y-Y-A-Q-L-Q-N-L-T-K-R-I	APC 150 - 169
M 4	L-Q-T-D-M-T-R-R-Q-L-E-Y-E-A-R-Q-I-R-V-A	APC 180 - 199

M 5	T-R-R-Q-L-E-Y-E-A-R-Q-I-R-V-A-M-E-E-Q-L	APC 185 - 204
M 6	Q-D-M-E-K-R-A-Q-R-R-I-A-R-I-Q-Q-I-E-K-D	APC 208 - 227
M 7	A-R-I-Q-Q-I-E-K-D-I-L-R-I-R-Q-L-L-Q-S-Q	APC 219 - 238
M 8	D-D-M-S-R-T-L-L-A-M-S	APC 328 – 338
M 9	K-E-A-R-A-R-A-S-A-A-L-H-N-I-I-H-S	APC 377 - 393
**M10**	**L-H-L-L-E-Q-I-R-A-Y-C-E-T-C-W-E-W-Q-E-A**	**APC 407 - 426**
M11	G-M-D-Q-D-K-N-P-M-P-A-P-V-E-H	APC 430 - 444
M12	H-Y-S-I-T-L-R-R-Y-A-G-M-A-L-T-N-L-T	APC 492 – 509
M13	E-D-L-Q-Q-V-I-A-S-V-L-R-N-L-S	APC 538 – 552
M14	S-V-K-A-L-M-E-C-A-L-E	APC 568 – 578
M15	E-S-T-L-K-S-V-L-S-A-L-W-N-L-S	APC 582 – 596
M16	L-A-I-I-E-S-G-G-G-I-L-R-N-V-S-S-L-I	APC 629 – 646
M17	L-T-I-V-S-N-A-C-G-T-L-W-N-L	APC 674 – 687
M18	K-M-I-A-M-G-S-A-A-A-L-R-N-L-M-A	APC 716 – 731
M19	E-D-T-P-I-C-F-S-R-C-S-S-L-S-S-L-S-S-A-E	APC 1265-1284
M20	E-D-T-P-I-C-F-pS-R-C-S-pS-L-S-S-L-S-S-A-E	APC 1265-1284
M21	E-D-T-P-I-C-F-S-R-C-pS-S-L-pS-S-L-pS-S-A-E	APC 1265-1284
M22	E-D-T-P-I-C-F-pS-R-C-pS-pS-L-pS-S-L-pS-S-A-E	APC 1265-1284
M23	E-T-P-L-M-F-pS-R-C-T-pS-V-S-S-L-D-S-F-E	APC 1379-1397
**M24**	**E-S-T-P-D-G-F-pS-C-S-S-pS-L-S-A-L-S-L-D-E**	**APC 1494-1513**
N 1	E-G-T-P-I-N-F-pS-T-A-T-pS-L-S-D-L-T-I-E-S	APC 1645-1664
**N 2**	**E-G-T-P-Y-C-F-pS-R-N-D-pS-L-S-S-L-D-F-D-D**	**APC 1850-1869**
N 3	E-N-T-P-V-C-F-pS-H-N-S-pS-L-S-S-L-S-D-I-D	APC 1957-1976
N 4	E-D-T-P-V-C-F-pS-R-N-S-pS-L-S-S-L-S-I-D-S	APC 2015-2034
N 5	E-D-T-P-V-C-F-S-R-N-S-pS-L-S-S-L-S-I-D-S	APC 2015-2034
N 6	G-G-G-H-H-H-H-H-H	Positive control

*ASAD bound to a peptide array of APC by the following peptides: APC$_{401–415}$, APC$_{407–426}$, APC$_{471–485}$, APC$_{1501–1515}$, APC$_{1494–1513}$, APC$_{1861–1875}$, APC$_{1850–1869}$ (bold).

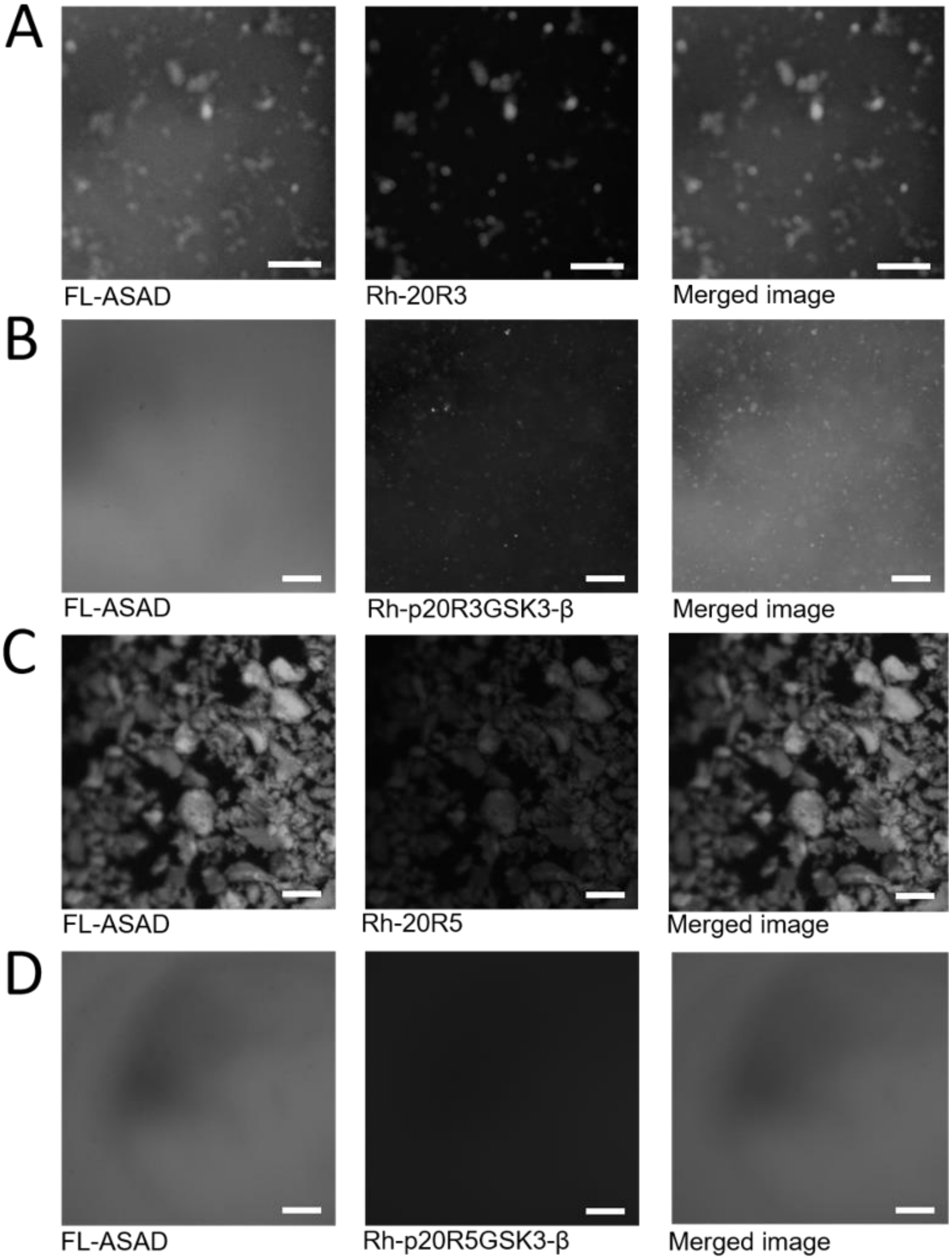

Figure S1. The effect of phosphorylation on droplet formation by APC derived peptides (gray scale copy of Figure 4): (**A**) ASAD labeled with fluorescein and 20R3 labeled with rhodamine b located to the same clusters (**B**) Phosphorylation of 20R3 prevented co-aggregation with ASAD. (**C**) ASAD labeled with fluorescein and 20R5 labeled with rhodamine b located to the same clusters (**D**) Phosphorylation of 20R5 prevented co-aggregating with ASAD. All scale bars = 20 μm.
